# Low Persistence of Inattention Symptoms From Childhood to Adolescence: A Population-Based Study

**DOI:** 10.1177/10870547231187147

**Published:** 2023-07-27

**Authors:** Astri J. Lundervold, Lin Sørensen, Maj-Britt Posserud, Mari Hysing

**Affiliations:** 1 University of Bergen, Norway; 2 Haukeland University Hospital, Bergen, Norway; 3 Regional Centre for Child and Youth Mental Health and Child Welfare, NORCE Norwegian Research Centre, Bergen, Norway

**Keywords:** adolescence, inattention, longitudinal

## Abstract

**Objective:**

To investigate the persistence of dimensional measures of inattention in a population-based, gender-balanced sample of adolescents with parent reports of inattention from childhood and self-reports of inattention in adolescence.

**Method:**

Adolescents, 16–19 years old, completed the Adult ADHD Self Report Scale. Their parents completed the Swanson, Nolan, and Pelham Rating Scale—4th Edition when they were 7–9 and 11–13 years old.

**Results:**

More severe inattention in boys than girls in childhood shifted to a female predominance in adolescence. The correlation between reports of inattention in childhood and adolescence was weak, with parent reports explaining only ~5% of the variance in the inattention score reported in adolescence.

**Conclusion:**

The weak association between inattention in childhood and adolescence is consistent with the emerging challenges associated with being an adolescent, but the impact of shifts in informants, gender and age on symtpom presentations should be investigated in future studies.

## Key points:

- There is a lack of studies on the symptom course of inattention in the general population.
- Inattention in a gender-balanced population-based study showed weak continuity from parent reports in childhood to self-reports in adolescence.
- Higher scores in boys than girls in childhood shifted to girls having the highest scores in adolescence.
- A more dynamic pattern with age- and gender-related changes should be considered when assessing and treating problems related to inattention.
- Further studies should investigate the impact of informant shifts from childhood to adolescence.

## Introduction

Inattention is one of the three core symptoms of Attention-Deficit Hyperactivity Disorder (ADHD), a neurodevelopmental disorder that tends to persist into adulthood (Biederman & Faraone, 2006). The persistence of inattention and other symptoms of ADHD has, however, recently been challenged by Moffit and collaborators (Moffitt et al., 2015), who suggested that childhood and adult ADHD should be described as non-overlapping diagnostic categories. Conflicting results are also reported in population-based samples. While some studies have reported persistence of parent-reported symptoms of inattention (e.g., Holbrook et al., 2016), others have rather reported a decrease in symptoms level with age (e.g., Dopfner et al., 2015). A recent study including a longitudinal ADHD sample rather showed that ADHD symptoms typically fluctuated across development (Sibley et al., 2022). By this, Sibley and collaborators emphasized the importance of accounting for the dimensionality of ADHD symptoms in longitudinal studies. The present study follows up on this finding by investigating a dimensionally defined measure of inattention in a population-based sample followed from childhood to adolescence. The importance of a wide and dimensional definition is underscored by results from studies showing that a substantial part of children (e.g., Polderman et al., 2007) and adolescents (e.g., Lundervold et al., 2016) in the population experience functional impairment due to an inattention below a diagnostic threshold (Zendarski et al., 2022).

Several factors may influence results in studies of inattention (Vos et al., 2022). First, there is often a need to change informant at different ages. Typically, parents report on inattention symptoms in childhood, whereas a shift to relying on self-reports happens in adolescence. Age is another influencing factor. Although inattention is reported as a problem across all ages, childhood inattention may have a more substantial impact on future functioning than inattention later in life (Ahmad et al., 2020; Daley & Birchwood, 2010; Farmer et al., 2002; Spira & Fischel, 2005). Inattention may for example prevent a child from learning basic skills, giving her severe problems when the curriculum becomes more complex at higher grade levels. This is supported by several population-based studies, showing that inattention is a strong predictor of academic grades at high school (Holmberg & Bolte, 2014; Lundervold et al., 2017; Pingault et al., 2014; Polderman et al., 2010). The importance of gender should be evident. A gender bias disfavoring boys in childhood (Cherkasova et al., 2022) shifting towards a more balanced frequency in adulthood (May et al., 2019) is well known from studies of clinical ADHD. This points to the impact of age and gender interactions. It has for example been shown that girls tend to show a severity level reaching clinical attention at a later age than boys (Murray et al., 2020). A tendency to leave inattentive girls undetected in childhood (American Psychiatric Association e.g., Shi et al., 2021) are plausible explanations of age-gender interactions. However, one should not downplay gender differences in adolescents due to the massive impact of interacting biological and environmental changes that occur in that period of life (Park & Chang, 2021).

Taken together, the literature referred to above motivated the present study to investigate associations between self-reported inattentive symptoms in a gender-balanced, population-based group of adolescents (16–19 years) who were rated by their parents when they were 7–9 and 11–13 years old. Inattention was defined according to the nine age-appropriate symptoms described in the Diagnostic and Statistical Manual of Mental Disorders (American Psychiatric Association, 2013) at all study waves. To perform a more detailed investigation of the most severe cases, we will also include analyses at a categorical level by defining a group of adolescents as inattentive. By this, we aim to contribute to the research field by focusing on inattention in a gender-balanced population-based sample, and by including age-appropriate items from the DSM to define inattention at different ages. The study will also shed light on the implications brought on by the necessary shift in informants from childhood to adolescence/early adulthood from parental report to self-reports.

## Methods and Measures

### The Bergen Child Study/Youth@hordaland Study

Data stem from three waves of the Bergen Child Study (BCS). The BCS was launched in 2002 by inviting parents and teachers of all children attending the second to fourth grade (7 to 9 years old) at any school in Bergen, Norway (study-wave 1 (W1), (Heiervang et al., 2007; Stormark et al., 2008). A second study wave (W2) was conducted approximately three years later when the children attended fifth to seventh grade (11 to 13 years old) (Boe et al., 2021). In a follow-up study, during the spring of 2012, all adolescents born between 1993 and 1995 living in the county including the city of Bergen were invited to participate (the youth@hordaland study). The present study included adolescents with parent reports on a questionnaire assessing inattention (see details below) when they participated in both the first and second study wave of the BCS. All adolescents in the county who attended high school received information via e-mail about the study, and one school hour was allocated for them to complete an internet-based questionnaire. Adolescents who were not in school received information by mail.

Age and gender of the participants were derived from the personal identification number from the Norwegian National Registry. The exact age was estimated by calculating the interval of time between the date of birth and date of participation. Socioeconomic status (SES) was assessed both by perceived economic well-being and parental education. Perceived economic well-being was reported with three response options: “poorer than others,” “equal to others,” and “better than others.” Maternal and paternal education were reported separately with three response options: “primary school,” “secondary school,” and “college or university,”

### Swanson, Nolan, and Pelham Rating Scale—4th Edition

To assess inattention in childhood, we included all items of the inattention subscale from the Swanson, Nolan, and Pelham Rating Scale—4th Edition (SNAP-IV) (Bussing et al., 2008). SNAP-IV is a widely used and psychometrically sound dimensional checklist of DSM-IV-defined symptoms of ADHD, including both an inattention and a hyperactivity/impulsivity subscale (Swanson et al., 2001). This scale has been widely used both in clinical and population-based studies, including the BCS. On the original form, the informant is asked to indicate on a four-point scale whether the behavioral descriptions fit the child “not at all,” “just a little,” “pretty much,” or “very much.” For the purpose of the BCS, this was altered to a three-point scale in order to ensure identical response categories for the entire questionnaire of the study (0 = “not true,” 1 = “somewhat true,” and 2 = “certainly true”). In the present study, we included dimensional information from SNAP-IV inattention and hyperactivity/impulsivity subscales as rated by parents in the first and second BCS study wave, where a higher score indicates a higher severity level. In that some studies argue for the impact of joint presence of inattention and hyperactivity/impulsivity in childhood (Leopold et al., 2019; Willcutt et al., 2012) on future function, parent reports of hyperactivity/impulsivity will be included as a control variable in some of our statistical analyses.

### Adult ADHD Self-Report Scale

The inattention subscale from the Adult ADHD Rating Scale (ASRS) was used to assess inattention in the adolescents (Adler et al., 2006). This scale includes items addressing the inattentive presentation of ADHD described in the DSM-IV diagnostic manual (Americal Psychiatric Association, 2013), and has been widely used both in clinical and population-based studies, including the young@hordaland study. For each of the items, the participants are asked to evaluate if they had *never, rarely, sometimes, often*, or *very often* experienced what is described by the text during the last 6 months. In the present study, a problem on a given item was defined as recommended by Kessler et al. (Kessler et al., 2005), where severe = “sometimes,” “often,” or “very often” response on items #1, 2, 3, 4, 9, and an “often” or “very often” response on all the other items. Severity level was defined as a continuous metric along the ASRS scale. Adolescents who reported severe problems on at least five of the nine ASRS items included in the inattention subscale were defined as inattentive.

### Analytic Process

Differences between males and females, between age groups, and the two severity levels defined from ASRS were explored using independent group T-tests or Pearson chi-square tests where appropriate. Bivariate correlation analyses were included to investigate associations between sum scores on the parent reports at the two time points in childhood and the ASRS scores in adolescence. Regression analyses were added to investigate the contributions of parent reports in childhood on the adolescents’ responses to the ASRS. Finally, we ran a subclass analysis focusing on individuals defined as inattentive in adolescence to investigate the accuracy of predictions from childhood reports.

### Ethical Considerations

The youth@hordaland study is recommended by the Regional Committees for Medical and Health Research Ethics in Norway (2011/811) and the Norwegian Agency for shared services in Education and Research. Inclusion was based on informed consent at each study wave.

## Results

### Description of the Sample

A total of 2177 adolescents participated in the study, 1230 girls. The mean age of the adolescents was marginally higher for girls (17.5 (.8)) than for boys (17.4 (.8, *p* < .05)). Almost all (99.1%) were high school students at the time of assessment. Very few adolescents reported that their mother’s education was at the lowest level (6.3%) and only 4.5% considered their family income as lower than in most families. A total of 242 adolescents were defined as inattentive according to self-reports on the ASRS, 66.6% girls. All adolescents included in the study were asked if they ever were given a diagnosis of ADHD or depression. Seven of the females and six of the males reported that they had a diagnosis of ADHD or ADD, and 22 (all girls) reported a diagnosis of depression.

### Inattention and Hyperactivity/Impulsivity Symptoms in the Three Waves

Table 1 shows that boys were reported by parents with a higher inattention score than girls, while the gender imbalance of severity level was negatively in the direction of girls on self-reports on ASRS. According to the effect sizes, the mean differences on ASRS were small and small to medium for parent reports on SNAP-IV. A similar gender-specific pattern was shown for hyperactivity/impulsivity, with small effect sizes in favor of girls in childhood, and with a small, but statistically significant more severe hyperactivity/impulsivity symptoms reported by girls than boys in adolescence.

**Table 1.** Wave Specific Inattention and Hyperactivity in Boys and Girls.

	All *n* = 2177	Boys *n* = 947	Girls *n* = 1230
W1 IN	1.67 (2.4)	2.13 (2.8)	1.31 (2.1)*** *d* = 0.33
W1 HI	1.22 (2.2)	1.57 (2.6)	0.95 (1.8)*** *d* = 0.28
W2 IN	1.8 (2.8)	2.4 (3.3)	1.35 (2.3)*** *d* = 0.37
W2 HI	0.80 (1.9)	1.09 (2.7)	0.58 (1.4) *** d = 0.24
W3 IN	14.9 (5.8)	14.0 (5.7)	15.6 (5.7)*** *d* = 0.28
W3 HI	11.6 (5.2)	11.1 (5.3)	12.1 (5.1)***, *d* = 0.19

*Note*. IN = Inattention subscale; HI = hyperactivity/impulsivity subscale; *d* = Cohen’s *d*; W1 = parent SNAP 7–9 years old; W2 = parent SNAP 11–13 years old; W3 = self-reports ASRS 16–19 years old; *d* = Cohen’s *d* = (M2–M1)/SD_pooled._

*** = *p* < .001.

### Correlations Between Reports in Childhood and Adolescence

Table 2 shows the bivariate correlations between the inattention and hyperactivity/impulsivity scores at the three study waves. The table shows strong correlations between parent reports of inattention in childhood, with a much weaker correlation with the adolescents’ self-reports both for boys (*r* = .22) and girls (*r* = .14). A similar trend was shown on the hyperactivity/impulsivity subscale, but we note a weaker correlation between the inattention and hyperactivity/impulsivity subscales across the two first waves for girls than for boys on parent reports. An analysis of the two self-reported ASRS subscales showed correlations at a moderate level both for girls (*r* = .60) and boys (.58).

**Table 2.** Correlations Matrix Between Inattention and Hyperactivity Symptoms, Presented Separately for Girls (in Red) and Boys (in Blue).

	W_1 IN	W_2 IN	W_3 IN	W_1 HI	W_2 HI	W_3 HI
W_1 IN	1	.63**	.16*	.60*	.49***	.07*
W_2 IN	.57**	1	22***	.47**	.64**	.11**
W_3 IN	.16 **	.14**	1	.09	.13***	.58**
W_1 HI	.56**	.40**	.12**	1	.68*	.13**
W_2 HI	.39**	.50**	.07*	.56**	1	.18**
W_3 HI	.15**	.12**	.60**	.16**	.14**	1

*Note.* Results for girls to the left, below the midline (red) and boys to the right, above the midline (blue). W_1 to W_3 = the three study-waves; IN = inattention; HI = hyperactivity.

* Significant at .05 level. **Significant at .01 level. ***Significant at < .001 level.

### Prediction of Self-Reported Inattention in Adolescence

Table 3 shows the results from the regression analysis when information about gender and parent reports in the first and second study waves were included as independent variables and the self-reports on ASRS as an outcome variable. In model one, gender explained 2% of the reports on the ASRS inattention subscale, with an add-on of around 3% when information about childhood inattention was included. When this analysis was run separately for boys and girls, parent inattention reports explained ~5 and 3% of the variance in the ASRS scores, respectively. Inclusion of information about hyperactivity did not add to the explained variance, neither in the whole group nor within either of the two gender groups (Table 3).

**Table 3.** Inattention Scores in Adolescence Explained by Childhood Reports.

	*B*	Beta	*t*	*p*	*r* ^2^	*r* ^2 change^	*F* ^change^
Inattention							
Model 1							
Gender	2.105	.180	8.47	<.001	0.020	0.020	<0.001
Inattention wave 1	0.188	.079	2.99	.003	0.044	0.024	<0.001
Inattention wave 2	0.271	.132	4.94	<.001	0.054	0.011	<0.001
Inattention and hyperactivity							
Model 2							
Gender	2.01	.180	8.45	<.001	0.020	0.020	<0.001
Inattention wave 1	0.183	.77	2.59	.010	0.044	0.024	<0.001
Inattention wave 2	0.294	.143	4.86	<.001	0.054	0.011	<0.001
Hyperactivity wave 1	0.033	.013	0.42	.674	0.055	0.000	0.987
Hyperactivity wave 2	−0.081	−.026	−0.86	.390	0.055	0.000	0.390

*Note*. B = Beta coefficient; Beta = standardized coefficient: confidence interval; *r*^2^ = *r*-square; *p*-value; *r*^2^ change = Change in explained variance; *F*^change^ = significant *F* change.

### Parent Childhood Reports in Adolescents Defined as Inattentive

A total of 242 adolescents were defined as inattentive (i.e., they reported severe problems on at least five of the nine ASRS items included in the inattention subscale; see Methods and Measures section), with a predominance of girls (67%). Only four of these participants reported a diagnosis of depression or ADHD. Diagnostic information was therefore not included in the ROC curve analysis.

The analysis showed an overall weak classification of the adolescents defined as inattentive versus non-inattentive based on parent reports from parents in childhood from both the first and second wave, a finding that was reflected by a weak AUC both for boys (.602 and .652) and for girls (.566 and .561).

## Discussion

The present study investigated symptom reports of inattention from childhood to adolescence. The results showed that the persistence was weak from parent reports of inattention in childhood to self-reports of inattention in adolescence, even when childhood reports were used as predictors of the most severe inattentive cases in adolescence. The correlation between parent reports in wave one and two was stronger, more so in boys than in girls. Overall, there was a shift from more severe parent-rated inattention in boys than girls in childhood to higher symptom scores reported by girls in adolescence. These findings were found in a large gender-balanced population-based sample.

Assessment of the DSM-defined symptoms of inattention at three time points makes the present study and its results somewhat different from previous studies. The present study was different by including questionnaires with the same number of items, with wordings of the items that were age-adjusted to measure the same class of behavior described in the DSM system. The results from this study were, however, weaker than expected from previous longitudinal studies including clinical samples (see e.g., (Cherkasova et al., 2022) and in studies where adults retrospectively described childhood behavior (e.g., Lundervold et al., 2020; Owens & Hinshaw, 2016). A stronger persistence should, however, be expected from population-based studies reporting that inattention in childhood impacts a wide range of future functions (Ahmad & Hinshaw, 2017; Holmberg & Bolte, 2014; Meque et al., 2019). More studies are indeed necessary before firm conclusions can be settled.

Although the persistence of inattention symptoms is weak, the high scores reported by adolescents and the high number of adolescents defined as inattentive are worth commenting. This probably reflects a substantial impact of newly emerging factors that occur during adolescence. Increased severity may for example be due to the high load on attention regulation experienced by high-school students, commonly combined with a stronger pressure on vigilance and reduced support from parents and teachers. Even though most adolescents obtained scores below the threshold for an ADHD diagnosis, they may still experience challenges corresponding to those reported in studies of diagnosed adolescents (Faheem et al., 2022; Franke et al., 2018). Combined with a weak persistence, our results gives support to what Park and Chung (Park & Chang, 2021) described as an “inverted U” shape of the trajectory of inattention. They showed a peak in inattention at age groups similar to the one used in wave 3 of the present study. This and the dynamic model presented by Sibley et al. (Sibley et al., 2022) for ADHD, indicating fluctuations at different ages, should be models to be tested in further longitudinal studies on inattention in a population-based sample.

As already stated in the introduction, the shift from parent reports in childhood to self-reports in adolescence may be critical both for the content and the thresholds of the items used to define inattention (Murray et al., 2022). An agreement between parents and self-reports from their child has been reported to be at a moderate to low level even when performed within the same time point (Hemmingsson et al., 2017; Meyer et al., 2022; van der Ende et al., 2012), and it is reasonable to assume differences in agreements across age groups. A weaker link between parent and child reports would, for example, be expected in adolescence than at a younger age when the child probably is more influenced by the comments and opinions of parents (van der Ende et al., 2012). Although a weak association thus could be expected even if both self-reports and parent reports were included at all three time points in the present study, this issue is indeed open for further investigation.

The present study contributed with some gender-specific results. Overall, they confirmed previous findings of a female predominance of symptoms related to inattention in adolescents (Lundervold et al., 2016). Although the association with parent reports in childhood was weak for both genders, it was somewhat stronger for the two childhood reports for boys than for girls. This is in line with studies showing that reports of inattention and other ADHD-related symptoms are skewed in disfavor of boys in childhood (Coles et al., 2012; Hinshaw et al., 2022). The weaker association for girls could indicate that parent reports are less reliable and valid for girls and that parents are less aware of the challenges as experienced by girls. This pattern of findings resonates with the gender bias seen in female ADHD, where girls are consistently referred later, diagnosed later, and less often offered adequate treatment, and that this occurs even though girls commonly experience significant problems like a high risk of self-harm and problems in close relations (Hinshaw et al., 2022). A better characterization of gender-specific inattention in childhood would thus potentially be important to elucidate targets for improving detection of and treatment options for girls and women. The importance of further studies adding emotional function to inattention was suggested by the present results, showing that a combination of self-reported ADHD and depression diagnoses was only found among the girls.

### Strengths and Limitations

The main strength of the present study was the inclusion of a large, population-based, and gender-balanced sample of adolescents with parent reports from two time points in childhood and one in adolescence, the use of the same DSM-defined measure of inattention across all three study waves, and by focusing on a symptom (i.e., inattention) that is both common and disabling both across psychiatric diagnoses (Carmichael et al., 2015) and in the general population of children (e.g., Polderman et al., 2007) and adolescents (e.g., (Lundervold et al., 2016).

Several limitations must be accounted for. The wide timespan is a strength but also introduces a selection bias, as only youth at high school with reports from the three time points were included. This bias is expected to underestimate the severity of inattention in the population by selecting for less inattentive youth (Stormark et al., 2008). The generalizability of the results may also be restricted by all adolescents having a childhood in the city or close surroundings to the city of Bergen. The number of adolescents reporting ADHD was also lower than expected from a previous publication from young@hordaland (Boe et al., 2016), probably due to inclusion restricted to high-school students. It should also be mentioned that a previous study from the Bergen Child Study/youth@hordaland has found that the responders for all three waves, were more likely to be female, have parents with higher education, and come from families with better financial circumstances than non-responders (Sivertsen et al., 2017).

The use of a single informant and a single measure at each time point may also have introduced a bias. With self-reports from adolescents, a response bias in the self-reports cannot be ruled out (see Mulraney et al., 2022). Challenges related to inclusion of different assessment tools and informants should be emphasized. Although there are arguments for using an age-unspecific assessment tool across different age groups, we decided to use two instruments that were age-adjusted, with items giving measures of overlapping inattentive behaviour. The adolescents were asked about diagnoses, but only information on ADHD and depression was included in the present study. Cultural differences should also be mentioned, in that some studies indicate culture differences in the presence and consequences of inattention (Ahmad et al., 2020).

## Conclusions

The present study contributes by investigating the persistence of inattention, measured with a wide and sound definition of inattention across three different time points in a population-based, gender-balanced sample. Persistence was found to be stronger in boys than girls according to parent reports in childhood, but overall weak from childhood to adolescence. Interestingly, among the most severe cases of inattention, there was a higher prevalence of adolescent girls than boys. A peak in severity of inattention in adolescence is suggested, and further prospective studies including a wider range of features are called for to validate the present findings and to make more accurate predictions of inattention in individual adolescents.
